# Studying Dynamics of Human Information Gathering Behaviors Using Social Robots

**DOI:** 10.3389/fpsyg.2021.669198

**Published:** 2021-06-01

**Authors:** Matan Eshed, Matan Epstein, Ada H. Zohar, Goren Gordon

**Affiliations:** ^1^Ruppin Academic Center, Hadera, Israel; ^2^Curiosity Lab, Department of Industrial Engineering, Tel-Aviv University, Tel-Aviv, Israel

**Keywords:** social interaction, dynamics of interaction, curiosity, psychological inflexibility, social robots, human-robot interaction

## Abstract

A novel social interaction is a dynamic process, in which participants adapt to, react to and engage with their social partners. To facilitate such interactions, people gather information relating to the social context and structure of the situation. The current study aimed to deepen the understanding of the psychological determinants of behavior in a novel social interaction. Three social robots and the participant interacted non-verbally according to a pre-programmed “relationship matrix” that dictated who favored whom. Participants' gaze was tracked during the interaction and, using Bayesian inference models, resulted in a measure of participants' social information-gathering behaviors. Our results reveal the dynamics in a novel environment, wherein information-gathering behavior is initially predicted by psychological inflexibility and then, toward the end of the interaction, predicted by curiosity. These results highlight the utility of using social robots in behavioral experiments.

## 1. Introduction

Human beings, like other organisms, are driven to reach, explore and engage with their surroundings by their very nature (Berlyne, 1950; Hebb, 1955; Spielberger et al., 1994). As observations on infants confirm, we are attracted to novel, ambiguous and complex stimuli by an intrinsic drive for self-expansion (Switzky et al., 1974; De Charms, 2013). Social interaction with other novel and lively agents manifests these attributes even more, due to the highly ambiguous nature of the other social agents, which possess their own differentiated will, and therefore can act in unexpected ways. Exploratory behaviors of humans and animals have been studied in the past in the context of approach and avoidance behaviors (Berlyne, 1954; Kreitler et al., 1974; Power, 1999). These behaviors were also examined as part of social interactions with humans and other life-like objects such as virtual characters and social robots (Satake et al., 2009; Leite et al., 2013). Less is known about individual psychological differences that influence behavior in a novel interaction, and especially those that influence the approach behaviors that will be selected by the individual. As De Jaegher et al. (2010) proposed, it is possible that the best way to learn about social cognition is through a deeper investigation of the interactions themselves.

### 1.1. The Use of Social Robots

In the current study we wish to utilize a dynamic systems tool approach for analyzing the structure of the interaction patterns, as suggested by De Jaegher et al. (2010). For this purpose, we have selected to use social robots as the social partners for the human participant. But can social robots be used for studying human psychological aspects? Several studies suggest that this is indeed the case.

In the context of trustworthiness, analyzing human non-verbal gestures enabled the programming of a humanoid robot to use similar gestures, resulting in similar human responses and perceptions (DeSteno et al., 2012). Using multi-modal human-human-robot interactions datasets for studying personality and engagement showed that using data from human-human interaction resulted in similar personality classification as human-robot interaction, suggesting that people exhibit similar personality-related behaviors in both scenarios (Celiktutan et al., 2019).

A large survey of non-verbal behaviors (Saunderson and Nejat, 2019), including robot gestures, proxemics, tactile interaction and time-experiencing interaction (e.g., hesitation) has shown that humans easily perceive these interactions and respond to them in similar ways they respond to humans performing the same actions.

Moreover, recent studies have shown that when robots behave socially toward other robots, their anthropomorphism increases, and when they behave socially toward people, participants are more willing to interact with them (Fraune et al., 2020). Thus, we assume that the person's exploratory tendencies in novel social interactions will be manifested also when interacting with partially inanimate agents, such as social robots (Auvray et al., 2009; De Jaegher et al., 2010).

While studies have shown that stress can affect the initial perception of social robot poses' valence and arousal (Thimmesch-Gill et al., 2017), a brief interaction with a social robot was shown to decrease uncertainty and increase reported social presence (Edwards et al., 2019), alluding to a rapid acceptance of social robots as social partners. Finally, social robots have been used to study human curiosity expressions (Epstein and Gordon, 2018).

Social robots have several compelling attributes that make them advantageous to human confederates in studying social interactions:

1. Social robots are *fully autonomous* agents with whom a person could socially interact.
2. They drastically *reduce potential noise factors* that exist in interactions with actual humans. More specifically, when dealing with the measurement of social traits, the experimenter who runs it and the confederates who take part, can pose considerable confounding factors.
3. They enable full *control over various interaction parameters*, such as non-verbal behaviors, social feedback and information content.
4. A completely autonomous experimental setup enables cleaner measurements of the human participant's behavior, as all *other factors are known and controlled* by the setup itself.
5. Social robots' behavior is completely repeatable and unwavering for the entire course of a long study, enabling more robust measurements of the *dynamical* nature of the interaction.

Hence, we have chosen to use social robots as the research tool, more specifically the NAO humanoid robot (Beck et al., 2010; Häring et al., 2011; Erden, 2013).

The aim of the current study is to deepen the understanding of the psychological factors that influence behavior in a novel social interaction. More concretely, these behaviors address inferring the attitudes of the social agents present in the social interaction toward each other and toward the participant.

The study covers two main psychological domains which are dominant in the context of novel environments: (1) curiosity and (2) psychological inflexibility. These domains will be examined through an interaction with social robots, where we chose to focus on non-verbal behaviors, to reduce the complexity of verbal communication and content-related biases.

### 1.2. Non-verbal Behaviors

Humans and social robots use non-verbal behaviors to communicate. However, since social robots offer full control over their poses and gestures, their perception by their human partners is of equal importance (Saunderson and Nejat, 2019).

Robot's use of gestures have been shown to have effects in the context of learning and memorization of story details (Bremner et al., 2011; Huang and Mutlu, 2013); increased collaborative task performance (Breazeal et al., 2005), and induce higher level of engagement (Sidner et al., 2005; Gielniak and Thomaz, 2012). Furthermore, it has been shown that non-verbal gestures are perceived more positively when combined with speech, compared to only speech (Aly and Tapus, 2013).

To better replicate human gestures, learning from demonstration has been used and has been shown to create the same valence perception as human gestures (Seo et al., 2015). Furthermore, parameterizing mood behavior in humanoid robots has also been shown to foster mood contagion of the human partner, exhibiting similar effects to interacting with humans (Xu et al., 2015).

It has been shown that specific gestures can communicate better, based on the appropriate scenario, for example, exhibiting (i.e. grasping and lifting an object) works better when initially the object is not in the line of sight of the listener, whereas presenting an object (gesturing towards an object with a full extension of the fingers) works well when the referrer is distant from the objects (Sauppe and Mutlu, 2014). Recently, a large database of gestures was collected for human-robot interaction (de Wit et al., 2020). However, these gestures were for specific task-related scenario, such as words gestures (Hayes et al., 2013; de Wit et al., 2020) or joint attention (Sauppe and Mutlu, 2014), but not non-verbal communication gestures that express attitudes.

Since the current study utilizes *only* non-verbal robot behaviors, it was imperative to fully describe and analyze the perceptions of the behaviors used (see Section 2).

### 1.3. Curiosity

Curiosity has been defined as the recognition, pursuit, and desire to explore novel, uncertain, complex and ambiguous events (Gordon, 2018; Kashdan et al., 2018). Other definitions include an approach-oriented behavior, a derivative of an inner motivation that makes us want to seek, gather and assimilate new information and experiences (Ryan and Deci, 2000). Several theories attempt to explain information seeking behavior, such as uncertainty reduction theory (Berger and Calabrese, 1975; Bradac, 2001) and information gap theory (Loewenstein, 1994). The latter resulted in both quantitative measurement tools (Jirout and Klahr, 2012) and the emergent field of curious robots (Gordon, 2020).

People with greater curiosity expand their psychological resources by engaging in activities that are personally and socially enriching (Silvia, 2006). Brain imaging studies have shown that induced “curiosity states” result in improved learning (Gruber et al., 2014). However, exploration occurs mainly when one feels secure (Ainsworth and Bell, 1970). Considering these facts, curious people are more likely to be more engaged in information-gathering behaviors during social interactions, once they are accustomed to them.

### 1.4. Psychological Inflexibility (PI)

Psychological flexibility is a complex term that captures a group of psychological traits and certain cognitive styles, which lead to dynamic processes that unfold over time and show the way people cope with daily experiences (Bonanno et al., 2004; Hayes et al., 2004). PI includes difficulty in adapting to new situations, and approaching them with anxiety and distrust. Psychologically flexible people can better adapt to changing situational demands, showing less rigidity in their cognitive style, relying less on heuristics and stereotypes and managing their mental resources and behavior (Kashdan and Rottenberg, 2010). One of the main building blocks of psychological flexibility is executive functioning (Kashdan and Rottenberg, 2010), i.e., the ability to inhibit dominant behaviors, shift between strategies and control attention. In a social interaction, a situation that may invoke also negative feelings, flexible people probably have more executive resources and hence can choose more freely to interact.

### 1.5. Dynamical Nature of Social Interaction

The aforementioned studies of curiosity and PI hint toward their influence on the dynamical nature of social interaction. Thus, for example, PI mainly manifests when something *changes* (Kashdan and Rottenberg, 2010), e.g., during the beginning of a new interaction. On the other hand, curiosity requires the subjective feeling of security to manifest (Ainsworth and Bell, 1970), which may occur after an initial interaction and not at the beginning of it (Edwards et al., 2019).

Moreover, high uncertainty (Edwards et al., 2019) and anticipation toward an *interesting novel event* increases learning (Gruber et al., 2014), thus may introduce an association between curiosity and learning only at the beginning of the interaction, and not after it has continued.

However, the direct quantitative investigation of social interaction dynamics and personality traits is still lacking.

### 1.6. The Current Study

Here, we considered a quantitative model-based approach to social interaction. Our model enabled us to dissociate information-gathering (IG) behaviors from learning. Here we define IG behaviors as *actions* that result in acquiring new information, and learning as *using the new information* to solve a task.

By tracking participants' gaze and using Bayesian inference models, we were able to compute a distance metric from the “optimal information gathering agent,” called Information-Gathering Behavior Error (BE). By directly asking the participants how they perceive the social structure, we were able to measure their learning. These behavioral and cognitive correlates enabled us to track the dynamic nature of a novel social scenario, and study the influence curiosity and PI may have on them. Based on the aforementioned literature, we hypothesize that:

(H1) There will be a positive association between curiosity and information gathering behaviors, with a stronger association later in the interaction, due to increased sense of security (Ainsworth and Bell, 1970) and decreased uncertainty (Edwards et al., 2019).
(H2) There will be a negative association between psychological inflexibility and information gathering behaviors, with a stronger association during the beginning of the interaction (Kashdan and Rottenberg, 2010).
(H3) There will be a positive association between curiosity and learning, with a stronger association during the beginning of the interaction (Gruber et al., 2014).

To run the study, we first had to create a social interaction among robots and a human participant that enabled a dynamical investigation of a social structure. In this human-robot-robot study, robots interacted with one another, as well as interacting with the human participant.

Hence, we first conducted an on-line study (“Preliminary Study”) to validate the non-verbal robot-robot behaviors and their perception by human observers. The study resulted in perceptual probability distributions that enabled us to use Bayesian models for the generation and assessment of non-verbal behaviors' attitude meanings.

We then conducted our main study to investigate how human participants explore and learn dynamical social structures, by viewing and participating in a human-robot-robot non-verbal social interaction.

## 2. Preliminary Study: Validation of Non-verbal Robot-Robot Expressive Behavior Repertoire

**Figure 1:**
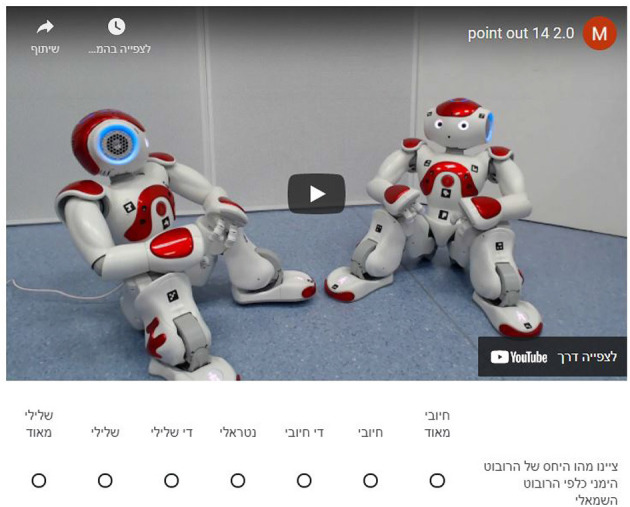
On-line questionnaire example (in Hebrew, the native language of the rates). The question reads: Please rate the attitude of the robot on the right to the robot on the left. The 7-point Likert scale ranges from “very negative” on the left to “very positive” on the right.

The preliminary study addresses the design of robot gestures toward other robots that can convey a large variety of attitudes' valence and ambiguity toward another agent. To validate these designs, we performed an on-line study with videos of robots gesturing toward one another and asked naive raters to rate their perceived attitudes' valence, Figure 1.

### 2.1. Methods

We used the NAO robot as the robotic platform for its humanoid form, its prevalence in human-robot interaction studies and its ease-of-use for generating gestures (Beck et al., 2010; Häring et al., 2011; Erden, 2013; Thimmesch-Gill et al., 2017; Marmpena et al., 2018). We created 16 different gestures using Aldebaran Choregraphe, based on the social meanings of nonverbal behaviors in humans (Rashotte, 2002), Figure 2. Naive raters were used to obtain ground truth for the valence that each robot gesture expresses. 16 short videos (15–30 s long) which present the gestures performed by one robot toward another were presented in a random order. Following each video, the raters were asked to answer a single question to indicate the attitude of the gesturing robot to the other robot (“Indicate what was the relation of the right robot toward to left robot”). The answer used a 7 point Likert-scale ranging from Negative to Positive (“Very negative,” “Negative,” “Pretty negative,” “Neutral,” “Pretty positive,” “Positive,” “Very positive”). There was no time limit for the questions and the raters could watch the video multiple times.

**Figure 2:**
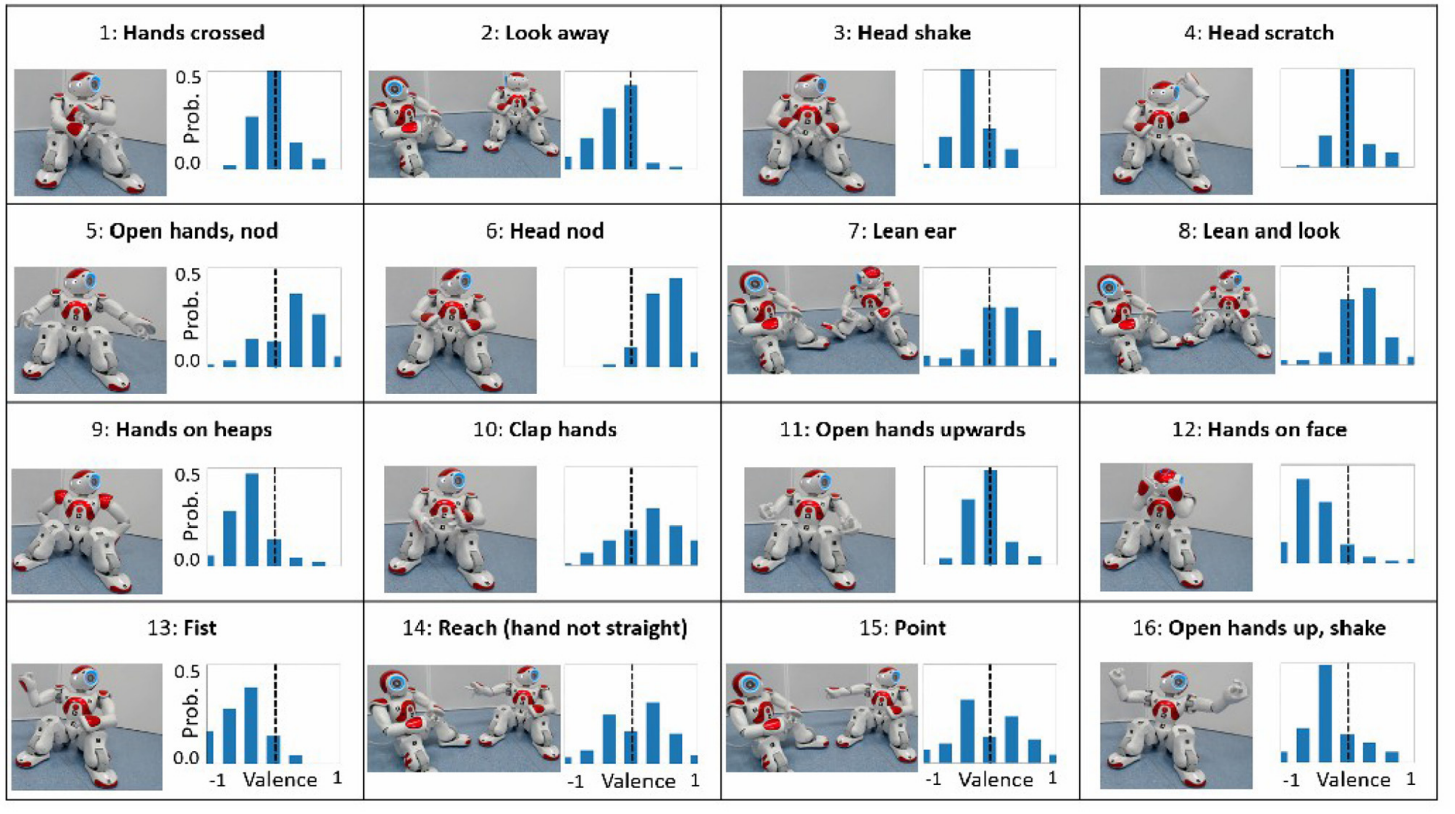
Robot gestures and attribute valence (X) probability (Y) distribution (two robots were always present in the videos).

To screen for raters that were not performing the task adequately, we performed two filters. We added a trap-question after the seventh video. In that video, it was written on the screen with bold letters exactly how to fill the following scale. We removed raters who did not answer the question correctly. Furthermore, we removed raters whose total time for the rating was under 180 s (which is the total minimal time it takes to watch all videos).

#### 2.1.1. Participants

One hundred and five naive raters were recruited from our university. The final data contained 94 participants, including 43 females and their average age was 28 (±7) years. They all signed a consent form and the study was approved by the Institutional IRB.

### 2.2. Results

We normalized the 7 point Likert scale to a range of [−1, 1] and then computed a histogram of ratings for each gesture. Dividing all the histogram data by the number of observations, resulted in the probability of perceiving the attitude's valence given the gesture.

From this probability distribution *p*(*v*), *v* ∈ [−1, 1], we extracted two important parameters: the expected value of the attitude, *A* = ∑*vp*(*v*), which accounts for the valence axis; and the normalized entropy of the distribution, *H* = ∑*p*(*v*)log_2_*p*(*v*)/*log*_2_(1/7), which accounts for the ambiguity of the gesture, ranging from *H* = 0 for a clear gesture, to *H* = 1 for a completely ambiguous gesture, where all attitude values have 1/7 probability.

**Figure 3:**
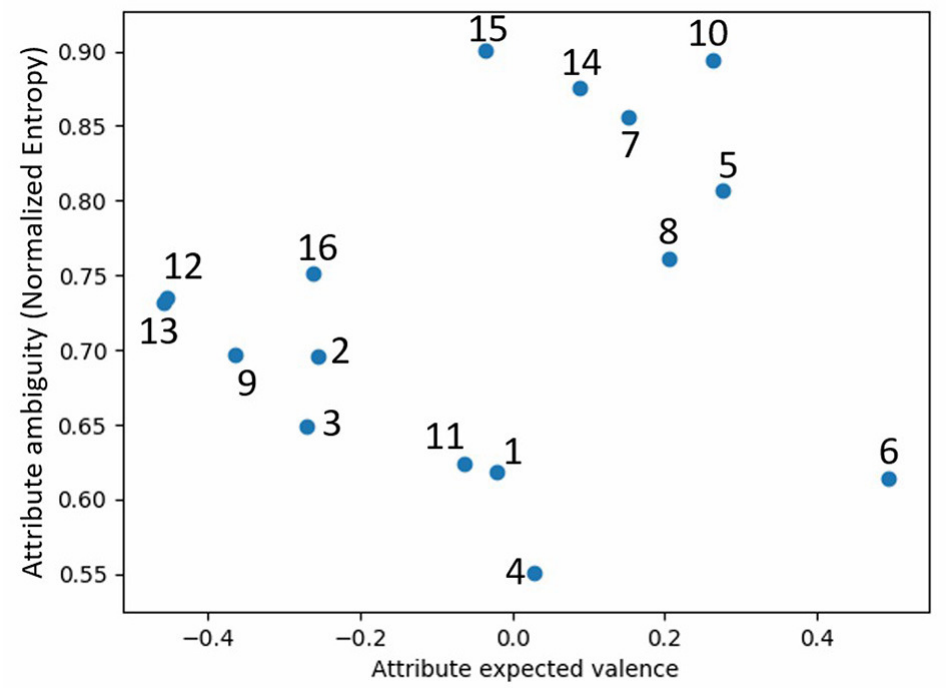
Robot gestures on the valence-ambiguity plane (gesture numbers refer to Figure 2).

Figure 2 shows the gestures and their probability distributions; Figure 3 shows their position on the valence-ambiguity plane. The 16 gestures (number in parenthesis) covered a large range of positive (6), neutral (1) and negative (13) attitudes. Furthermore, there was also a large variability in clarity of gestures, as is evident from the spread across the entropy axis, from clear gestures (4) to almost completely indiscernible gestures (14).

### 2.3. Discussion

While the preliminary study has involved videos and an on-line survey, and not live interactions with robots, this methodology has been used extensively in the human-robot interaction community, also to explore non-verbal interactions (Erden, 2013; Hayes et al., 2013; Sauppe and Mutlu, 2014).

We have based the 16 robot gestures on the social meanings of non-verbal behaviors in humans, reported in Rashotte (2002). While the list of behaviors were stated to convey different attitudes on the positive-negative axis, we have shown that there is also an important aspect of ambiguity (Sauppe and Mutlu, 2014). In contrast to previous studies, which have performed extensive research on emotional expression of *individual* robots (Erden, 2013; Marmpena et al., 2018), we have focused on interpersonal gestures, between two robots. Thus, we introduced new important gestures, such as pointing, nodding, leaning, that have a different meaning in a singular scenario. Furthermore, the videos presented *only robots*, meaning the participants experienced our desired scenario of seeing and rating robots communicating between themselves.

We have found that robot gestures of attitudes do relate to human gestures (Rashotte, 2002), in contrast to automatic gesture recognition, as reported in Hayes et al. (2013). Thus, nodding (5, 6) and leaning toward the other robot (7, 8) were perceived as positive gestures, whereas aggressive (13) and shy/embarrassed (12) gestures were perceived as negative. Moreover, some gestures were clearly neutral, such as head scratching (4), whereas other gestures, such as pointing (15) and reaching (14), were perceived very differently by various people. Future studies should also consider the cultural background of the participants, as the valence of a given gesture may be perceived differently by people from other cultures.

Compared to previous studies of emotional expressions of robots, which resulted in a list of gestures per emotional state (Beck et al., 2010; Häring et al., 2011; Erden, 2013), this study has resulted not just in the perceived aspect of each gesture, but also a quantitative probability distribution of its perception (Marmpena et al., 2018). This can be used not only in understanding how people perceive robots' gestures, but also to generate appropriate gestures based on required attitudes, while maintaining variability and engagement. Based on this analysis, we have discarded gestures 7, 10, 14, 15 due to their high ambiguity, and used the other gestures' probability distributions in Study 2 to generate a random gesture based on required attitude.

## 3. Main Study: Information Gathering Behaviors and Learning

### 3.1. Overview

In this study we addressed the issue of human-robot-robot interaction in a complex, fully autonomous setup with four Nao robots and a single human participant. An important aspect of our study is the (almost) non-existent intervention of humans. One Nao robot was the experimenter, and aside from the human experimenter greeting the participants into the room toward the setup and calibrating the sensors, all the rest of the study, including instructions and clarifications, was performed by the Nao experimenter. This resulted in an all-robot experimental setup, thus eliminating any human-related biases. The study itself was composed of three Nao robots non-verbally communicating toward one another and the human participant, using gestures from the preliminary study and a Bayesian-based algorithm. The goal of the participant was to infer the “relationship,” i.e., attitudes, of the robots toward one another. In other words, the human participants were part of an all-robot social scenario, which they had to decipher. We used eye-tracking to measure which robot the person was looking at, which in turn dictated which robot gestured next. Hence, while the human participants perceived the robot-robot social interaction, their gaze had influenced the interaction itself. In this way, the interaction and the information supplied was reciprocal, such that the human participant was also part of the flow. The interaction repeated several times, in order to measure any learning effects the participants may have in inferring the attitudes the robots conveyed toward each other.

### 3.2. Experimental Setup

A video presenting the experimental setup is available here.

**Figure 4:**
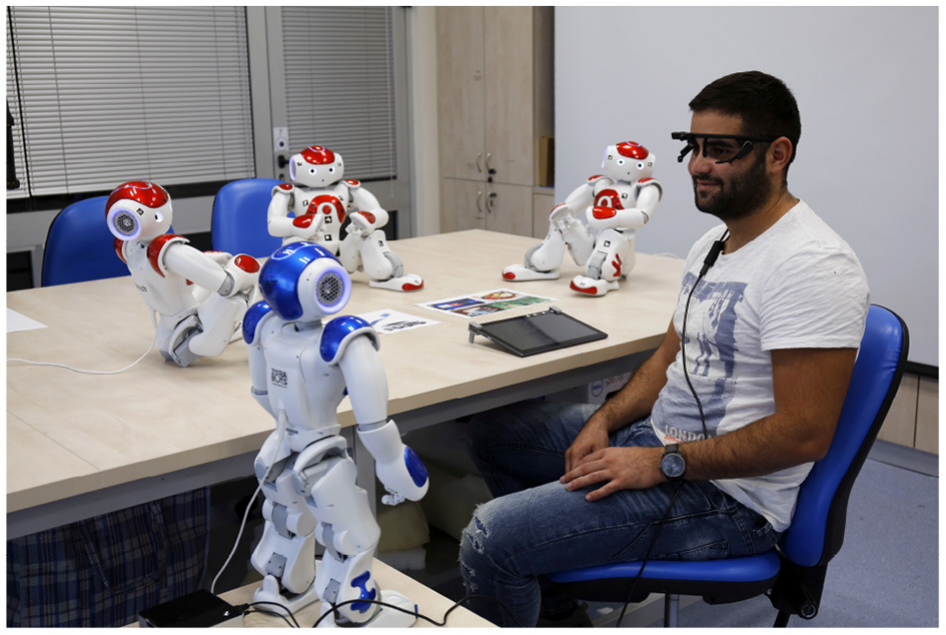
The experimental setup.

**Robots**: The experimental setup consisted of four fully autonomous NAO robots, a tablet and a Pupil eye tracking sensor (Kassner et al., 2014), programmed via ROS and Python. The participant sat in front of a table where three NAOs were positioned so that they were conducting a non-verbal interaction. The tablet was placed on the table in front of the participant and the fourth NAO stood to the left of the participant and served as the (robot) experimenter, Figure 4. The (human) experimenter sat behind the participant, out of his line of sight and monitored the interaction, but did not interact with the participant.

During the experiment, the robots socially interacted among themselves and with the participants. This conversation was a “silent” conversation which meant that the communication between the robots was based only on non-verbal cues (gestures) (Burgoon et al., 2016).

**Relationship matrix**: A relationship matrix was configured for each round of the experiment that described the relationship between every two entities (robot → robot / robot → human participant). The matrix dimensions represent the relationships which we can define—the three robots (3) relate to each other and to the human (4) (we cannot define how will a participant relate to the robots)—thus a (3*X*4) matrix was created, where the diagonal of the matrix is set to zero (since the robots do not relate to themselves). The matrix values, *M*_*i,j*_ ranged from 0 to 1 (with 9 discrete values), where 0 reflects a strong negative relationship or attitude, from *i* to *j*, and 1 represents a strong positive relationship from *i* to *j*. Each experiment round used a different matrix to define the relationship. In order to create matrices which are identical in their learning complexity (how hard it will be to learn them) we used a permutation of [0.27, 0.61, 0.94, 0.16, 0.94, 0.05, 0.5, 0.72, 0.5] in order to create all matrices. e.g., one permutation of these parameters can generate the following matrix:

(1) $$\begin{array}{l} M_{i,j}=\left(\begin{array}{cccc} 0 & 0.50 & 0.72 & 0.50\\ 0.61 & 0 & 0.27 & 0.05\\ 0.94 & 0.16 & 0 & 0.94 \end{array}\right) \end{array}$$

We chose five matrices out of the many permutations in the following way: First, to create realistic matrices, we eliminated all permutations that created matrices where ∣*M*_*i,j*_ − *M*_*j,i*_∣> 0.25, i.e., the difference between the way two robots feel about one another cannot be larger than $\frac{1}{4}$ in the scale of relationships. Second, we classified each matrix to the binary relationship it represents, i.e., which robot received the highest “aggregated attitude” from the other robots and which received the lowest aggregated attitude from the other robots. For example, for the relationship matrix shown above the aggregated attitude is [1.55, 0.66, 0.99] and the binary relationship it represents is [1, −1, 0], i.e., the first robot received the most positive attitude, the second the most negative and the third in the middle. Finally, we chose from each 6 binary relationship representation the matrix which has the highest aggregated attitude standard deviation, thus taking from each family of possible relationships, the matrix that will be the easiest to learn.

**Robot gestures**: The robot gestures were taken from the preliminary study, i.e., we took the probability distributions from Figure 2, which gave us the probability of a relationship given a gesture. Using conditional probabilities and the probability distribution computed above, we calculated the probability to choose each gesture based on the relationship parameter—the relationship to gesture probability database [RGPD].

We programmed the robots, using the naoqi API and ROS, to move in a life-like manner using two schemes. First, based on eye blinking research (Bentivoglio et al., 1997; Yoshikawa et al., 2007) we configured the robots to blink with a time between blinks drawn from an exponential distribution with: λ = 3.5*s* in normal mode and λ = 2.5*s* when the robot's relationship to the interaction subject was >0.5, thus expressing an increased blinking for positive attitudes.

Second, the robots moved their heads in a life-like way by performing random micro-movements with a velocity and range that changed depending on the robot's relationship toward the other participants (robot/human) that interacted with them, thus expressing a slower and smaller range movement for positive attitudes (Hadar et al., 1985).

**Sensors**: For the purpose of knowing where a participant is looking, we used the Pupil Labs eye tracker. Using the pupil capture software and by placing markers on the robots, we configured each robot as an identifiable surface, thus we knew in real-time where the participants were looking and if they were looking at one of the robots.

This setup enabled us to assess the participants' learning and exploration behavior when introduced to a novel mapping, in repeated rounds.

### 3.3. Protocol

The protocol was composed of three separate sections, namely, pre-study (at home), preparation and study.

#### 3.3.1. Pre-study

Forty-eight hours prior to the experiment, participants got via email a link to fill out an online questionnaire, which included a consent form. At the end of this questionnaire, they got a unique code which they brought with them to the experiment.

#### 3.3.2. Preparation

After signing another consent form, related to the study itself, and validating the home questionnaire code, participants were presented with general information about the experiment stage: an interaction with robots, a cognitive task and in the end, filling out several more questionnaires. Calibration for the Pupil eye tracking was done using the manual marker calibration method, and then participants were seated next to the table with the other robots. Once done, the experimenter told the participants that in this part of the experiment (interacting with the robots) the robot to their left (the blue robot) will give all the instructions and will act as the experimenter, and when the experiment starts they will be notified by the robot experimenter. The (human) experimenter said he had to complete some administrative tasks and *did not instruct* the participants at all.

#### 3.3.3. Study

**Figure 5:**
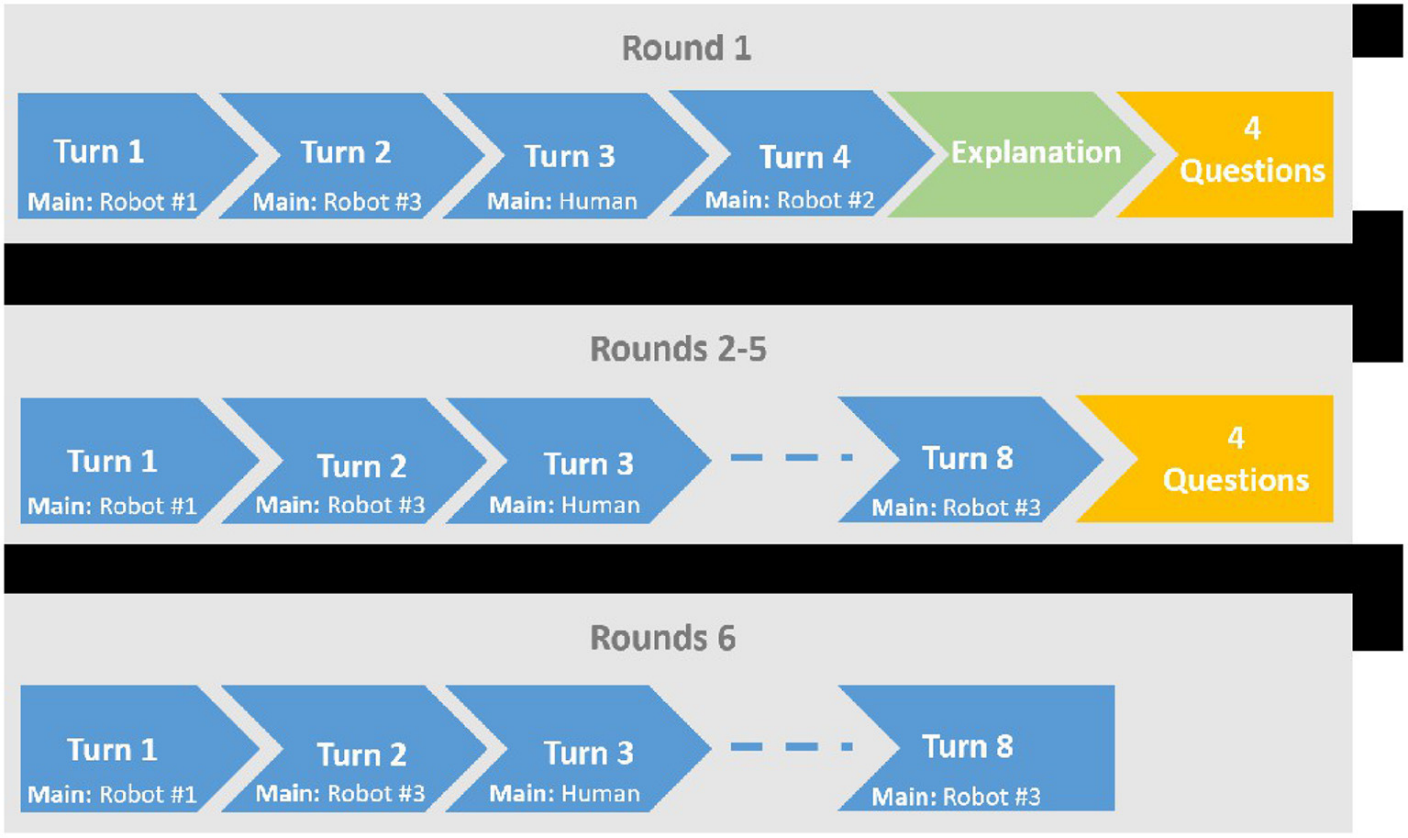
Main study protocol.

The study was based on human-robot-robot interaction, which was composed of 6 rounds, Figure 5. Each round was composed of a varied number of turns which lasted about a minute. We chose this setup for the interaction since we saw it enabled us to collect sufficient data without exhausting the participants. In each turn, a different robot (dubbed “the main robot” for this turn) gestured expressively toward one of the other robots, conveying a non-verbal message. In response, the other two robots used gestures in order to convey their matrix-based relationship toward the main robot. The main robot was preselected for every round. A unique turn also occurred, in which all three robots looked at the participant, in expectation for her movements. After a 5 s long interval, each of the three robots behaved according to their attitude, given by the current round's relationship matrix and the RGPD.

In every round there were three types of turns: (i) Initial robot turn; (ii) Human turn: the “unique” turn where the human participant was the main character. This kind of turn occurred once or twice in each round, in preselected turns and; (iii) Dynamic main robot turn: for the other turns in each round [those in which neither (i) nor (ii) were preselected], the main robot was selected based on the participant's behavior in the prior turn. Based on the pupil eye tracker data we calculated which robot (but not the main robot) got the maximum attention from the participant (i.e., the participant looked at it for the longest accumulated time). This robot was the next turn's main robot. This was done for two reasons, namely, to simulate an interaction where attention creates action and to give the participant control over the information she was going to receive next.

The first round of the interaction started immediately, where the robots performed a non-verbal interaction using gestures from Study 1 and based on their relationship matrix. Thus, initiating the first assessment of unprompted exploration, namely, what will participants do when not instructed to do anything, yet the robots interacted in front of them. We decided on having the first round without giving the participants explicit instructions in order to simulate the scenario where a human encounters several robots interacting, without knowing their mode of interaction.

During the first, taskless round, there were four turns, where the third was the Human Turn. Afterwards, the robots stopped the interaction, but still seemed alive—using blinks and micro-movements of the head, and the experimenter robot started a social interaction by explaining what is going on: “Hello, how are you? I'm Roby and I'm running the experiment. Before I start to explain the experiment, I do not know if you noticed, but while you were waiting, there was a social interaction between the robots. I will now ask you some questions about the interaction. Please answer using the tablet, and if you do not know the answer, that's fine.”

This introduction was followed by the robot asking four questions:

1. Which robot demonstrated the most positive attitude (relative to the rest of the robots)?
2. Which robot demonstrated the most negative attitude? (to the other robots)
3. Which robot demonstrated the most positive attitude towards you?
4. Which robot demonstrated the most negative attitude towards you?

The participant then answered the questions using a tablet, which presented the three robots and an option to select “I don't know” as the answer.After answering each question, the experimenter robot gave a feedback based on the participant's answer: “Well done, excellent” and “Good work, you recognized correctly”—for a correct response; “Not true, but nice try” and “Wrong answer, but that's OK, it's hard”—for an incorrect response. The three robots also conveyed this feedback, wherein the correct answer's robot raised its hand and the other two robots looked at it. Inquiring the participants about the interaction constituted our measure of learning, i.e., did the participant learn the relationship matrix and can she answer questions which require aggregated data from that matrix.

The first round was followed by the experimenter robot's explanation: “That's it, we've finished the questions. So let's start, I'll explain to you the course of the experiment. This part of the experiment is divided into several rounds. On each round, the robots and you will have a “silent” conversation. At the end of the interaction, I will ask you questions, as I did before. Some important points: Note that the relationship between the robots themselves and between the robots and you varies from round to round. The conversation is mute, which means that only body language will be used. Last thing, in every turn of the conversation, the next robot to talk is the robot that got the most attention from you.” In some cases participants requested to hear the instructions again, since they were not listening the first time due to possible excitement or inattention. Therefore we decided to play the instructions twice for all participants.

Following this explanation, the participants took part in four more rounds of interaction, each consisted of eight turns and the same four questions at the end of each round. During each round, a new relationship matrix was used.

The participants then took part in the sixth and last round which was not limited by time. In this last round, the participants were instructed as follows: “You may now interact with the robots for as long as you want. Please let the experimenter know when you are finished. You *will not* be asked any questions after this round ends.”

To summarize, the participants took part in six rounds: An initial round with no instructions, 4 rounds with instructions, and a last “free exploration” round. These six rounds constitute the basis for the analysis of perception of attitudes and dynamics of learning. The entire interaction was done in Hebrew, the participants' native language, and the average interaction length was about 20 min.

The participants then filled out several questionnaires using Qualtrics and performed the WCST computerized task (see below).

### 3.4. Instruments

#### 3.4.1. Questionnaires

All questionnaires were translated from English into Hebrew and then back-translated twice by two independent English-Hebrew bilinguals. Cronbach's Alpha values for the current study's sample are noted.

**Psychological Inflexibility**: *AAQ-2*: Participants completed the Acceptance and Actions Questionnaire-II, which measures psychological inflexibility (Bond et al., 2011). It includes 10 items on a 7-point Likert scale from 1 = “never true” to 7 = “always true” (α = 0.87). A higher score indicates that a person is less flexible.

**Curiosity**: *CEI-2*: Participants completed the Curiosity and Exploration Inventory II (Kashdan et al., 2009), which is composed of two subscales: stretching (α = 0.79) and embracing (α = 0.74) and a total score scale (α = 0.83). There are 10 items with 5 for each subscale scored on a 5-point Likert scale from 1 = “very slightly” to 5 = “extremely.”

**Human-robot interactions**: *NARS*: Participants completed the Negative Attitude toward Robots Scale (Nomura et al., 2006), which helps determine whether the participant has a general pre-existing negative attitude toward robots. The scale is composed of three subscales: Negative Attitudes toward Situations and Interactions with Robots (NARS “situations,” α = 0.76), Negative Attitudes toward Social Influence of Robots (NARS “social,” α = 0.73), Negative Attitudes toward Emotions in Interaction with Robots (NARS “emotions,” α = 0.76) and a NARS total score scale (α = 0.86). Altogether there are 14 items on a 5-point Likert scale from 1 = “strongly disagree” to 5 = “strongly agree.”

*Godspeed*: Participants completed the so called “Godspeed” scale (Bartneck et al., 2009), in which the participant reports about his perception of and feeling about the robots after the interactions. The scale is composed of 5 subscales: Anthropomorphism (α = 0.78), Animacy (α = 0.83), Likeability (α = 0.86), Perceived Intelligence (α = 0.65) and Perceived Safety (α = 0.77, after the removal of item number 24). Participants responded to 24 items on a 5-point semantic differential scale.

#### 3.4.2. WCST Task

Participants completed a computerized version of the Wisconsin Card Sorting Task (Heaton, 1981) provided by Inquisitlab. The sorting task includes 128 trials that are divided into two blocks of cards (two decks of cards). Each card represents three categories- shape, color and number, and every group of consecutive cards has at least one category in common. In every trial the participant was required to sort a single card into one of four piles of cards by a given rule (the common category), but every few trials the rule changed (the rule was set to change after 10 consecutive rounds in which the participant sorted correctly). The participant was given a feedback after every round (“Right” or “Wrong”) and had to try to match as many cards as possible while adjusting to the changing rules. In order to succeed in the task, it is necessary to inhibit the automatic response, and to shift the cognitive set in order to avoid perseverative and rigid responses. These abilities are part of the executive functions, and as already mentioned, psychological flexibility is strongly associated with executive functions (Kashdan and Rottenberg, 2010). The WCST was used to objectively assess psychological inflexibility. The sum of perseverative errors (sum PE) was calculated from the task data. A higher score on this measure indicates that a person is *less* flexible.

### 3.5. Data Analysis

All scales were calculated with the Python Pandas package, and all analyses were executed with SPSS®v22.

We have used the eye-tracking data to extract quantitative model-based measures of information gathering behavior. We describe below the data processing, calculations, and measures we have used in the study.

The Pupil Labs eye tracker sent the tracking information at 30 frames-per-second. From these data, we analyzed whether the participant was gazing at a robot at that time and if so, we logged it as *x*_*t*_ ∈ {1, 2, 3}, i.e., which robot the participant looked at, at time *t*. The data were segmented to each round, and within each round- to turns. During each turn, we flagged each data point if, at the time that the participant was looking at the robot, the robot conveyed information regarding the relationship matrix, i.e., performing an informative gesture.

Thus, our basic data structure for each participant *i*, round *s* and turn *z* was a sequence of robots *x*_*t*_ that the participant looked at, and the flag *f* ∈ {0, 1} which represents if this look was informative or not $\left(x_{t}^{i,s,z},f_{t}^{i,s,z}\right)$.

From each round, we extracted the following measures: We calculated the optimal estimation of the relationship matrix, based on the sequence of tracked gazes performed by the participant. This was done by applying Bayes' theorem and the given RGPD. In other words, we first defined a probability distribution over all possible relationship values, for each element of the relationship matrix, *p*_*j,k*_(*v*), where *v* ∈ (0, 1). Starting with a uniformly distributed probability distribution for each element, whenever the participant looked at a robot that expressed its relationship via a gesture *g*, i.e., not the main robot, we updated the appropriate probability distribution based on Bayes theorem: *p*_*j,k*_(*v*|*g*) = *p*_*j,k*_(*v*)*p*(*g*|*v*)/*p*(*g*), where the likelihood was taken from the RGPD. To obtain the participant's relationship matrix we calculated the expectation values: ${\tilde{M}}_{j,k}=\sum vp_{j,k}\left(v\right)$. Thus, for each subject and round, we obtained a matrix trajectory with each tracked gaze at time *t* = 1, ..., *T*, ${\tilde{M}}_{j,k}^{i,s,t}$.

Given two matrices, we defined their distance via:

(2) $$\begin{array}{l} d\left(M^{\left(1\right)},M^{\left(2\right)}\right)=\underset{j,k}{\sum }||M_{j,k}^{\left(1\right)}-M_{j,k}^{\left(2\right)}||/12 \end{array}$$

The real matrix is henceforth defined to be *M*.

The participants' behavior, i.e., their directed gaze, is thus influencing directly the dynamic nature of their matrix. This, together with the definitions above, enabled us to define the information-gathering behavioral error measure, labeled by us as “BE”:

(3) $$\begin{array}{l} BE_{i}^{s}=\left(1/T\right)\underset{t}{\sum }d\left({\tilde{M}}_{i}^{s,t},M_{i,global}^{s,t}\right), \end{array}$$

where $M_{i,global}^{s,t}$ is the *greedy global-optimal* matrix at each point in time. It is calculated by selecting the best gaze, at each point in time, starting from the first gaze. In other words, throughout the entire round, which robot will give the most information in each point in time. The $BE_{i}^{s}$ is a measure of distance from global optimality.

While the BE measure relates to the chosen *behavior*, we defined a measure relating to the *learning* process. At the end of the first five rounds, participants were requested to answer four questions. The answers were of a categorical type, and there was only one right answer for each question. The participants were requested to enter their answers into a tablet. In the tablet screen, a schematic picture of the three robots that corresponded to the way they were situated was shown, and the participants entered their answer by selecting one of the robots, or by choosing an “I don't know” option. After they entered their response, the participants got immediate feedback from the experimenter robot, indicating whether they were right or wrong. In total, each participant could get a score of 0–4 for each of first five rounds, and a maximum of 20 correct answers for the whole interaction. The sum of correct answers, which was labeled by us as “L,” represented to what extent the participants learned the pattern of relationships between the social robots; a larger L indicated that the participant learned more during the interaction.

### 3.6. Participants

Based on the most relevant prior research (Epstein and Gordon, 2018), we estimate correlations on the order of *R*^2^ = 0.15. For typical values of α = 0.05 and β = 0.2 (Type-II error), the required sample size is 62.

We first ran a pilot study, with 38 participants, to calibrate the experimental setup. We then conducted the main part of the study, with 84 participants. They were offered a payment of *$*30 for a total of 1 h that included questionnaires and a short interaction with social robots, and could register themselves via online platform. Participants were screened by language and previous participation in similar studies in the lab. Proficiency in Hebrew was required since the instructions were given in Hebrew, and since all of the questionnaires were translated into Hebrew. Participants who already took part in a previous lab's study could not participate in the current study, since it was important that they would not be able to guess the study's aim.

Participants from both studies, pilot and main one, (*N* = 122) completed the entire study, including filling up the questionnaires, completing the computerized version of the Wisconsin Card Sorting Test (WCST) and interacting with the robots. Two participants did not complete the WCST task.

For the instruments' analysis, we included participants from both studies (*N* = 122; Females: *N* = 80, Age: *M* = 26.2, *SD* = 6; Males: *N* = 42, Age: *M* = 25.8, *SD* = 5.9). Although the advertisement was not limited to students alone, most of the participants were undergraduate students from Tel-Aviv University, Israel (*N* = 106, 86%), who attended four different faculties (37% Exact sciences; 25% Social sciences; 13% Humanities and 11% Medicine). As participants were also asked whether they were diagnosed with ADHD, 13% of them reported positively (officially or self-diagnosed).

Analysis of the interaction with the robots included participants only from the main study (*N* = 84). Twenty-five participants were excluded due to technical difficulties: 4 participants did not take part in the interaction due to network communication problems; 16 participants had only partial eye-tracking data, which was only found after the study was finished; 5 participants encountered various other technical issues, such as power shortages etc. In total we had reliable data from 59 participants (Females = 37, Age: *M* = 27.3, *SD* = 8.1; Males: *N* = 22, Age: *M* = 25.3, *SD* = 5.1). The majority of these participants were students (*N* = 47, 79%), which came from four different faculties (23% Exact sciences; 34% Social sciences; 20% Humanities and 23% Medicine). 18.7% of the participants reported that they had been diagnosed with ADHD (officially or self-diagnosed). The descriptive data is summarized in Table 1.

**Table 1:** Sample properties.

	**Age (M)**	**Age (SD)**	**%Males**	**%Females**
122^*a*^	26.1	5.9	34.4	65.6
59 ^*b*^	26.5	7.1	37.3	62.7

a *Total participants, from pilot and main studies*.

b *Participants without technical issues*.

All participants signed a consent form and the study was approved by the Institutional IRB.

## 4. Results

The study results can be divided into two main groups of measures: internal experiment measures of the participants' performance in the interaction with the robots; and external measures, which include the self-report questionnaires and the WCST task. All measures' descriptive statistics are reported in Tables 2, 3. General correlations analysis between the external measures are reported in Table 4.

**Table 2:** Internal measures (N = 59) means (M) and standard deviations (SD).

**Measure**	**M**	**SD**
BE1	0.016	0.012
BE2	0.011	0.009
BE3	0.009	0.009
BE4	0.012	0.011
BE5	0.020	0.015
BE6	0.017	0.013
L1	1.470	1.194
L2	2.240	1.006
L3	2.460	0.934
L4	2.290	1.084
L5	2.680	1.074

*BE, Behavioral Error measure; L, Learning measure, with the numbers referring to the rounds*.

**Table 3:** Means (M) and Std. Deviations (SD) of the external measures and demographics.

**Measure**	***N*** **= 59**		***N*** **= 122**	
	**M**	**SD**	**M**	**SD**
AAQ^*a*^	2.5	.99	2.6	.99
WCST sum PE^*b*^	6.8	2.8	7.1	2.5
CEI total score^*c*^	3.4	0.6	3.5	0.6
CEI embracing^*c*^	3.1	0.7	3.2	0.7
CEI stretching^*c*^	3.8	0.7	3.8	0.7
NARS total score^*d*^	2.6	0.6	2.6	0.6
NARS emotions^*d*^	2.8	0.9	2.87	0.87
NARS situations^*d*^	2.3	0.7	2.3	0.7
NARS social^*d*^	3.0	0.8	2.9	0.77
Godspeed safety	3.3	0.6	3.10	0.7
Godspeed likeability	3.4	0.7	3.3	0.8
Godspeed animacy	2.8	0.7	2.8	0.78
Godspeed antro.^*e*^	2.5	0.8	2.4	0.8
Godspeed int.^*e*^	3.1	0.6	3.1	0.6
GPA	77.2	26.2	80.8	19.2
PET score	589.4	210.4	627.5	153.38

a *Acceptance and Actions Questionnaire-II*.

b *WCST, Wisconsin card sorting task; PE, perseverative errors*.

c *Curiosity and Exploration Inventory-II*.

d *NARS situations, Negative Attitudes toward Situations and Interactions with Robots; NARS social, Negative Attitudes toward Social Influence of Robots; NARS emotions, Negative Attitudes toward Emotions in Interaction with Robots*.

e *Antro., Antropomorphism; Int., Intelligence*.

**Table 4:** Correlations of PI measures with curiosity (*N* = 122)^*d*^.

	**CEI stretching^c^**	**CEI embracing^c^**	**CEI total^c^**
AAQ^*a*^	−0.164	−0.09	−0.142
WCST sPE^*b*^	−0.115	−0.037	−0.084

a *Acceptance and Actions Questionnaire-II*.

b *WCST, Wisconsin card sorting task; sPE, sum of perseverative errors*.

c *Curiosity and Exploration Inventory-II*.

d *All of the correlations above were not found significant, p > 0.05*.

General descriptive statistics (Table 3) show little difference between the two samples (*N* = 59 vs. *N* = 122) regarding the measures' averages and variability.

The internal measures are represented by two main variable groups: BE measures which stand for the participants' behavior (behavioral error) as it was measured by eye movement detection, and L measures which stand for the participants' learning (correct answers) as it was measured by the participants' answers to the questions that were asked after each round. Since the interaction was composed of six consecutive rounds, each internal variable was divided into six sub-variables: BE1 to BE6, and L1 to L5 (there is no L6, since the last round did not include any questions). Since our main hypotheses involve dynamics of novel social situations, we first analyze the dynamics of the interaction itself. The dynamics of learning and behavioral measures are analyzed separately first in Section 4.1, followed by analyses of their correlates to personality measures in Section 4.2. Implications of using social robots are analyzed in Section 4.3.

### 4.1. Dynamics of Behaviors and Learning

**Figure 6:**
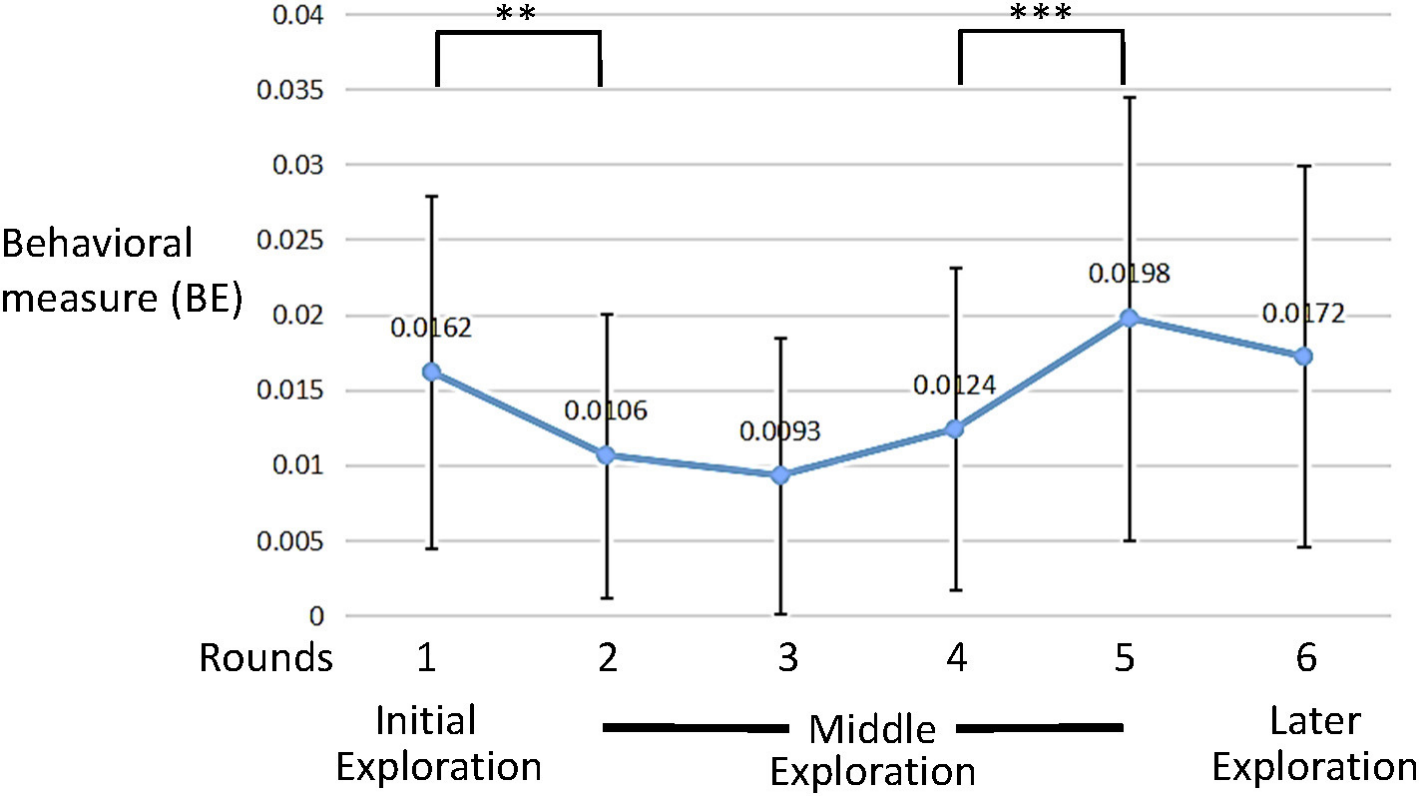
Average behavioral error (BE) measure values across trials (error bars denote standard deviation). **p = 0.002, ***p = 0.001.

**Figure 7:**
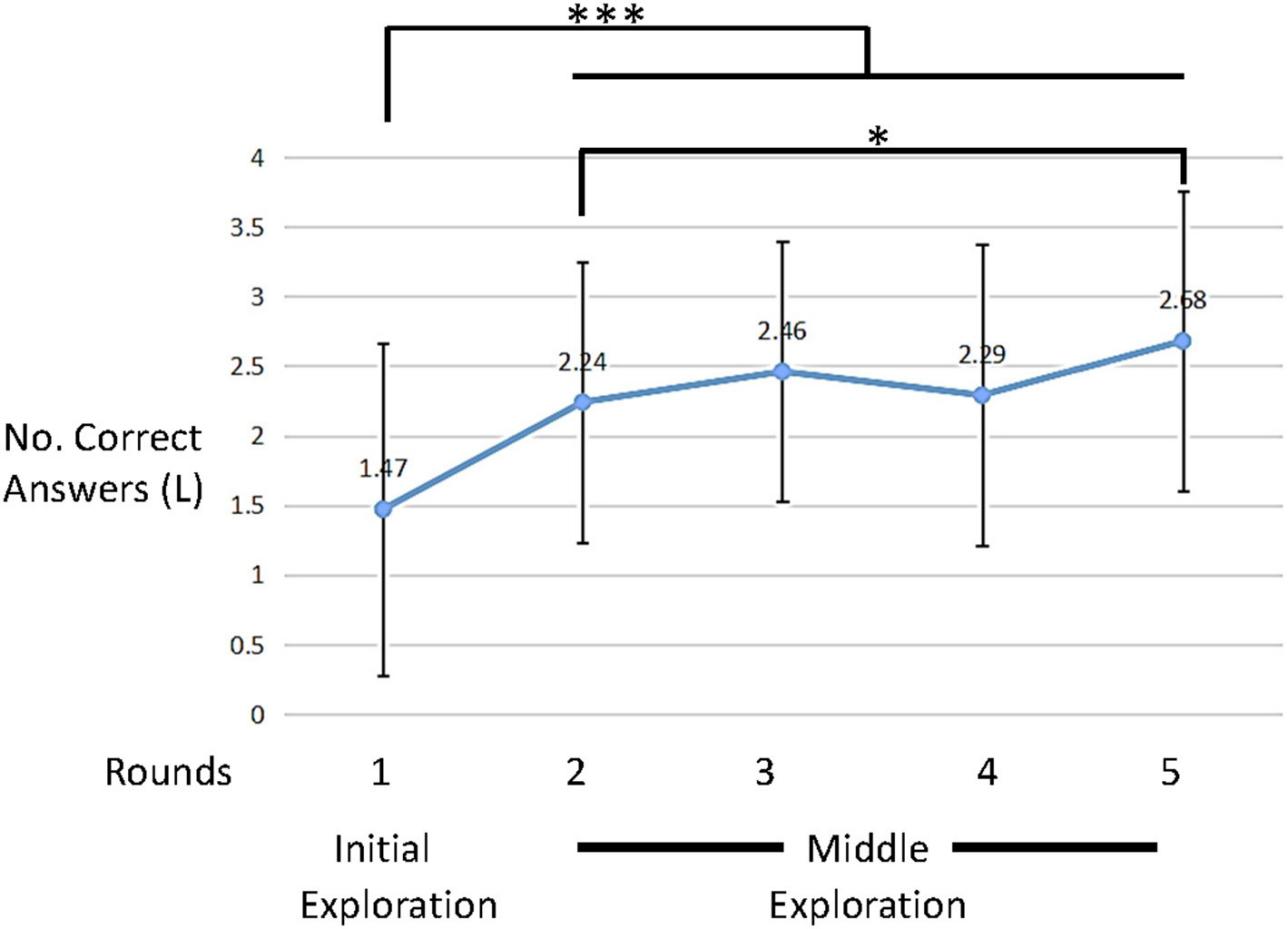
Average learning measure (L) values across trials (error bars denote standard deviation). *p < 0.05, ***p < 0.001.

As can be seen in Figures 6, 7, the distinctions between the six rounds of the interaction were in changes of both behavioral (BE) and learning (L) measures' average values. The six rounds could be divided into three sections: initial exploration (round 1), exploration at the middle (rounds 2-5) and later exploration (round 6). These data gave us a first glimpse at the participants' dynamics throughout the interaction.

A significant difference in the participants behavior was observed between round 1 and round 2 [BE: *t*_(58)_ = 3.30, *p* = 0.002, *d* = 0.429, paired *t*-test]. At the middle section (rounds 2–5) there were no significant changes in the participants' BE measures. However, a specific significant change was observed between rounds 4 and 5 [BE: *t*_(58)_ = 3.36, *p* = 0.001, *d* = 0.437, paired *t*-test].

With respect to learning, or completing the task, the first round, in which the participants were not aware of the task at hand, shows no learning as it was not-significantly different than random choice [*t*_(58)_ = 0.9, *p* = 0.367, one sample *t*-test].

However, Figure 7 clearly shows a significant learning trend as a function of rounds [$F_{\left(3.48,201.9\right)}=12.6,p<0.001,\eta^{2}=0.177$ ANOVA with repeated measures with a Greenhouse-Geisser correction]. Indeed the first round, unsurprisingly, shows significantly lower learning than the consecutive four rounds [*t*_(58)_ = 4.5, *d* = 0.597, *t*_(58)_ = 4.8, *d* = 6.3, *t*_(58)_ = 3.9, *d* = 5.1, *t*_(58)_ = 6.4, *d* = 8.9, *p* < 0.001 for pairwise comparisons of round 1 and rounds 2–5, respectively]. Moreover, while rounds 2–4 do not show a significant increase, there is a significant increase from round 2 to round 5 [*t*_(58)_ = 2.33, *p* = 0.023, *d* = 0.3 pairwise comparison]. Finally, rounds 2–5 show significantly higher learning compared to random choice [*t*_(58)_ = 6.9, *d* = 0.9, *t*_(58)_ = 9.2, *d* = 1.2, *t*_(58)_ = 6.7, *d* = 0.9, *t*_(58)_ = 9.6, *d* = 1.25 and *p* < 0.001 for rounds 2, 3, 4, and 5, respectively, one sample *t*-test].

**Figure 8:**
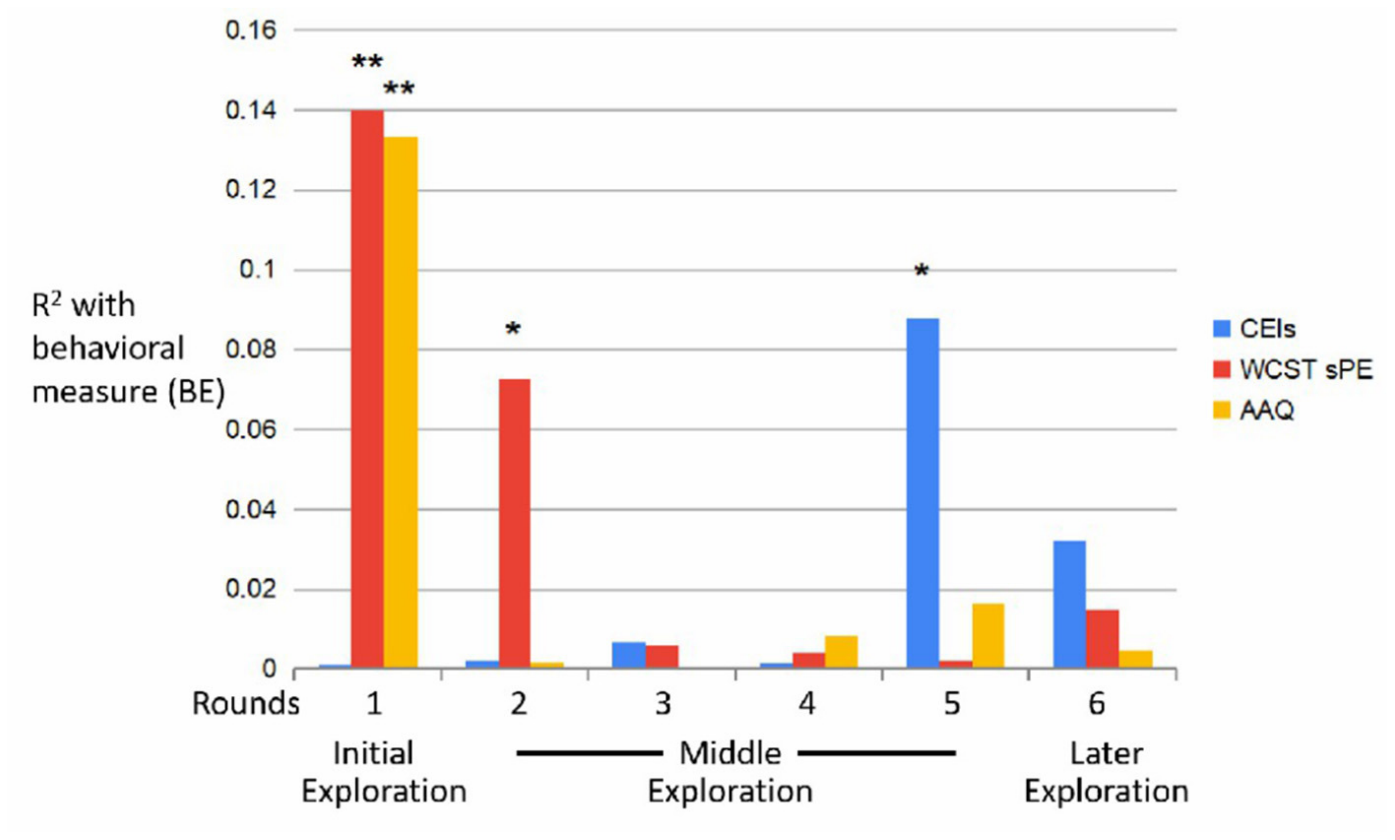
*R*^2^-values (BE) across trials. *p = 0.02 (CEIs), *p = 0.04 (sPE); **p = 0.004.

These results show that participants both improved in the task (Figure 7), but also changed their behavior (Figure 8).

### 4.2. Dynamics of Correlates to Personality Measures

**Figure 9:**
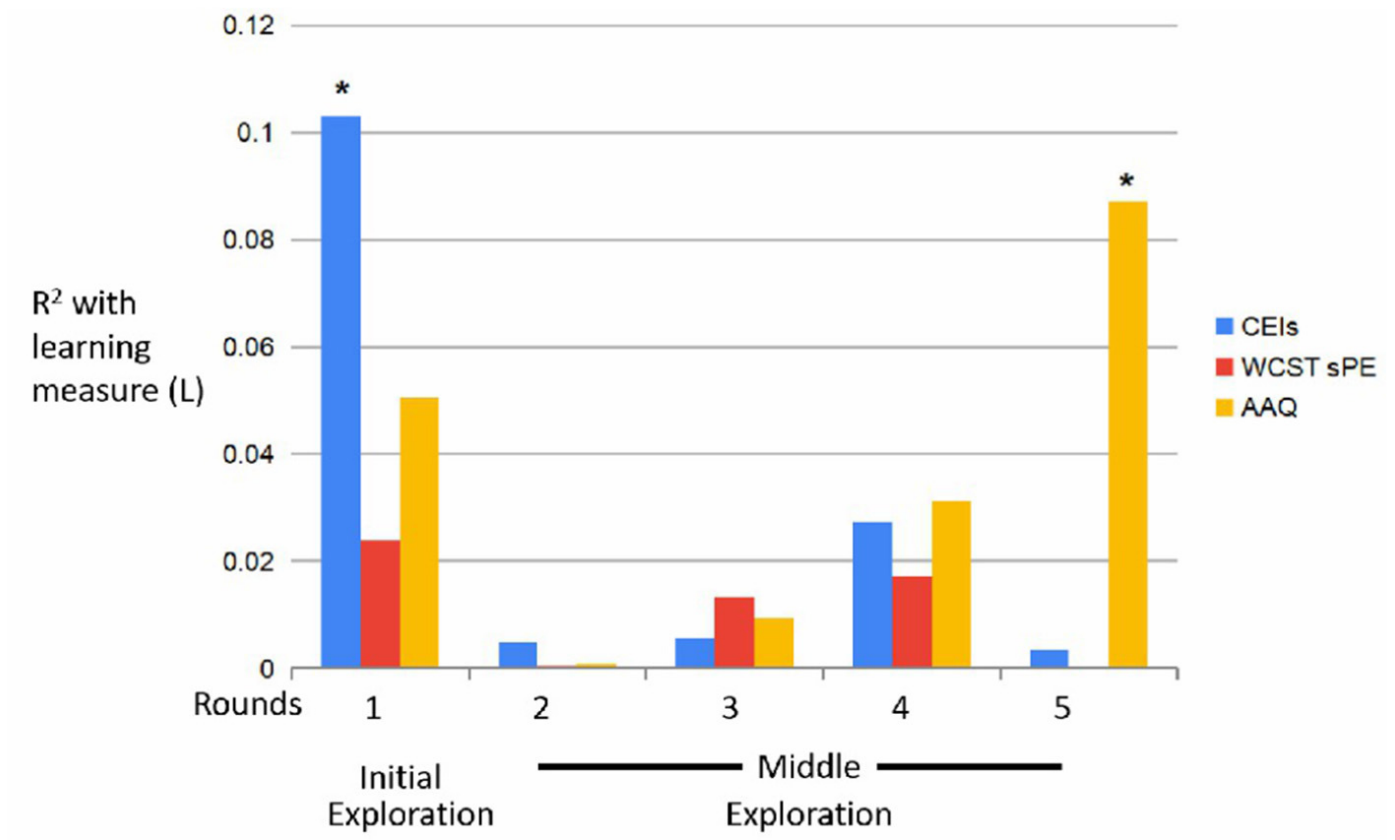
*R*^2^-values (L) across trials. *p = 0.01 (CEIs); *p = 0.02 (AAQ).

When inspecting the associations between the behavioral and learning measures, and the external measures, clear correlations could be found reflecting the dynamics of the internal measures (Figures 8, 9).

Results of a Pearson correlation indicated that there was a significant negative association between the CEI measure and the fifth round's behavioral measure (BE5 & CEI: *r*_(59)_ = −0.29, *p* = 0.02). This result supports hypothesis H1.

Results of a Pearson correlation indicated that there was a significant positive association between the sum PE measure on both the first (BE1 & sumPE: *r*_(59)_ = 0.37, *p* = 0.004) and second (BE2 & sumPE: *r*_(59)_ = 0.27, *p* = 0.04) behavioral measures. These results support hypothesis H2. However, results of a Pearson correlation indicated that there was a significant *negative* association between AAQ measure and the first round's behavioral measure (BE1 & AAQ: *r*_(59)_ = −0.36, *p* = 0.004), thus not supporting hypothesis H2.

As for the learning measures, results of a Pearson correlation indicated that there was a significant negative association between CEI and the first round [L1 & CEI: *r*_(59)_ = −0.32, *p* = 0.013], i.e., participants who self-report being more curious, were *less* correct during the first round, thus refuting H3. Moreover, while not significant, a positive association between self-report curiosity and learning appears in the fourth round [L4 & CEI: *r*_(59)_ = 0.129, *p* = 0.329, Pearson correlation].

Results of a Pearson correlation indicated that there was a significant positive association between the fifth round's learning measure and AAQ [L5 & AAQ: *r*_(59)_ = 0.295, *p* = 0.024].

Finally, the within-subject (repeated measures) test results revealed no significant interaction between the participants' sex or age and the internal measures (BE and L) in all of the interaction's rounds.

### 4.3. The Nature of Interacting With Robots

In the experiment we wanted to present the participants with a novel social interaction. Yet, it cannot be ignored that the interaction involved robots and not humans, which have unique properties that do not exist in other social interactions. In that sense, the robots could have served as a potential confound. Therefore the following analyses tested whether the unique perceived properties of the robots had any effect on the interaction.

In order to test whether the fact that robots were used has any significant influence on the interaction, the associations between the external robot associated measures (NARS and Godspeed questionnaires) and the internal robot interaction measures were directly tested. In that way we could get a preliminary picture of the amount of influence that these measures had on the internal measures. When the robot questionnaires were put together as predictors of the internal measures in a single model, the model was found significant only for L2 [*F* = 3(8, 50), *p* = 0.007, *R*^2^ = 0.32, *adj*.*R*^2^ = 0.22] when the Godspeed anthropomorphism scale was found to be a significant predictor (β = −0.44, *t*_(49)_ = −2.1, *p* = 0.039) and so were the NARS emotions scale [β = −0.52, *t*_(49)_ = −3.4, *p* = 0.001] the NARS situations scale [β = 0.54, *t*_(49)_ = 3.2, *p* = 0.002] and the NARS social scale [β = −0.52, *t*_(49)_ = −2.8, *p* = 0.007]. The model accounted for a total of 32% of the variance of L2. This finding suggests that the attitude toward robots was meaningful especially regarding the participants' learning at the beginning of the interaction, after the participants became aware of the fact that they will be inquired about the robot's interrelations following each round.

In addition, the associations between PI and curiosity and the internal measures were tested for moderation effects by the robot associated measures. The HC3 heteroscedasticity consistent standard error and covariance matrix estimator were used (Long and Ervin, 2000). We sought to find the most prominent effects the robots had on the predictive power of each group of independent variables. The results show that only PI measures were significantly affected: The sum PE measure was affected by the Godspeed intelligence and by the NARS social scales as a predictor of behavioral error (BE1). The AAQ scale was affected by the NARS social scale as a predictor of learning (L5). Data for the interaction coefficient, the yielded *R*^2^ change and the interaction direction is provided in Table 5 (only the significant effects are reported).

**Table 5:** Moderation effects by the robot associated measures (NARS and Godspeed).

**Category**	**Independent**	**Dependent**	**Moderator**	**β**	**F**	***R*^2^**	**Δ*R*^2^**	**Effect type**
PI	AAQ^*a*^	L5	NARS social^*b*^	−0.24^*^	3.4 (3,54)	0.14	0.05	L/+
	sum PE^*c*^	BE1	NARS social^*b*^	0.33^**^	15.8 (3,54)	0.24	0.1	H/+
	sum PE^*c*^	BE1	Godspeed int.^*d*^	−0.16^***^	32.6 (3,54)	0.19	0.045	L/+

* *p = 0.013, 95% CI[−0.4,−0.05]*;

** *p = 0.02, 95% CI[0.05,0.51]*;

*** *p = 0.02, 95% CI[−0.35,−0.02]*.

*H/+, higher moderator values imply a significant positive association; L/+, lower moderator values imply a significant positive association*.

a *Acceptance and Actions Questionnaire-II*.

b *NARS social, Negative Attitudes toward Social Influence of Robots*.

c *Wisconsin card sorting task; PE, perseverative errors*.

d *Int., Intelligence*.

In sum, the data shows that the NARS social and the Godspeed intelligence measures were the most prominent moderators. The NARS emotions and the Godspeed likability, animacy and safety had only minor effects. Secondly, the NARS measures tended to affect the associations at higher scores (e.g., when robots were perceived more negatively) and similarly the Godspeed measures tended to affect them at lower scores (e.g., when robots were perceived less positively). Thirdly, the robot associated measures did not significantly moderate the curiosity measures (CEI).

## 5. Discussion

In the current study, we investigated the determinants of human information-gathering behavior in a novel social interaction. We hypothesized that curiosity and psychological inflexibility would have a significant influence. As revealed by the results, our hypotheses were partially confirmed.

### 5.1. Roles of Curiosity and Psychological Inflexibility as Predictors of Learning Dynamics

The results suggest that the social task was indeed learnable, yet not implicitly so. We have found that the first taskless round was not significantly different than random selection, but participants improved in the task with continuing rounds. These unsurprising results set the stage for the non-trivial relation between self-report curiosity, as measured by the Curiosity and Exploration Inventory-II questionnaire (CEI), and learning the task. We found that there is a significant *negative* association between self-report curiosity and learning during the first round. This may be due to the novelty effect as reported by Smedegaard (2019), wherein participants were excited about the new situation they encountered, where more curious people experiencing this with a stronger magnitude. Other studies have reported a negative association between novelty and learning, especially so with social robots (Gordon et al., 2015).

An interesting association between PI, measured by the Acceptance and Actions Questionnaire-II questionnaire (AAQ), and learning was revealed. This *positive* association “rumps” up during the interaction, culminating in a significant positive association during the last round. This suggests that a more rigid approach to solving the social task is beneficial.

### 5.2. Roles of Curiosity and Psychological Inflexibility as Predictors of Information-Gathering Behaviors

We found that both curiosity and PI had significant associations with the participants' information-gathering behavior, although only at specific times during the interaction. Our results show an opposite trend between self-report PI (assessed by the AAQ questionnaire) and objectively measured PI (assessed by the WCST task's sum PE measure). A general significant positive association was revealed between participants with higher objectively measured psychological inflexibility and high behavioral errors at the beginning of the interaction, whereas an opposite association was found between their self-report PI. This finding is partly consistent with hypothesis H2. It partly corroborates our notion that flexible people can handle novel situations better than inflexible ones, and novel social interactions in particular. The effect of psychological flexibility was dominant at the beginning of the interaction with the robots, when the participants had to gather their emotional regulation and cognitive capabilities in order to fully invest themselves in becoming familiar with a novel and complex situation. As the participants became familiar with the interaction, the effect of psychological flexibility decreased.

However, contrary to PI, curiosity was found to be most meaningful at the end of the interaction and not at the beginning (Ainsworth and Bell, 1970). This finding is consistent with hypothesis H1 and with the early research on curiosity conducted by Hebb (1955) and Berlyne (1954). According to their model of curiosity, the association between curiosity and arousal could be described by an inverted U-shaped function, meaning that the level of curiosity increases as the level of arousal increases, but that it also decreases if the arousal level is too high. This is consistent with our finding that psychological inflexibility was dominant at the beginning of the interaction, which implies that the participants experienced relative high levels of anxiety at that time. And indeed, if the anxiety levels were too high at the beginning of the interaction, it means that the arousal level was also too high for curiosity to take place as a motivational factor. Apparently, a habituation period was needed before the participants could freely explore the situation.

### 5.3. Opposite Patterns of the Behavioral-Error and Learning Measures

The distinct trends between the Behavioral-Error (BE) and Learning (L) measures, and their personality-based correaltions indicate a substantial difference between them, which was at the level of consciousness to which they addressed: the participants were probably unaware of the eye tracking device during the interaction, which determined the behavioral measure as an implicit measure. On the other hand they were going through an explicit process when they were answering the questions that followed each round of the interaction, which determined the learning measure as an explicit measure. The differences between explicit and implicit learning were widely researched in the cognitive psychology domain (Reber et al., 1980; Willingham and Goedert-Eschmann, 1999; Batterink et al., 2015).

### 5.4. Study Limitations and Future Work

The current study aimed to investigate *human* information gathering behavior, using *social robots*. While several studies have shown that humans can treat social robots as they do humans (DeSteno et al., 2012; Celiktutan et al., 2019; Saunderson and Nejat, 2019), one cannot ignore this possible confound. However, our analysis of the effects of robot-specific attitudes and perceptions (NARS and Godspeed, respectively) shows that their effect is relatively small, occurs mainly in the beginning of the interaction (Edwards et al., 2019) and does not affect expressions of curiosity.

Furthermore, we have used non-verbal gestures, whose valence was quantified in the preliminary study, but also blinking and head movement, to convey positive or negative relationship. While we believe the effects of blinking and head movements were relatively minor, the distinct effect of each specific gesture on the interaction, as well as the direct effect of blinking and head movements, was left for future work. Integrating verbal communication, with nuances of speech intonation and emotional complexion was also beyond the scope of the current study.

The social robot experimenter framed the other robots' behaviors as “social interaction” in its introductory explanation. This may have primed the human participant to a specific perceptual path. Previous studies have shown that priming for curiosity, for example, can influence learning (Sher et al., 2019). Future studies can investigate framing of the interaction and its effect on information gathering behaviors.

Finally, the experimental setup was not 100% robotic, since the human experimenter still introduced the setup and remained in the room during the study. This may have had an effect, which will be studied in future work to better understand the implications of a truly full robotic experimental setup.

## 6. Conclusions

Curiosity and psychological inflexibility did manage to predict the extent to which the participants manifested information-gathering behavior during the interaction, but only at specific times. While psychological inflexibility was found to be a significant predictor at the beginning of the interaction, curiosity was found significant toward the end of it. These findings emphasize the complexity of social interactions, even when they are held with robots, and revealed possible dynamics of human exploration and engagement.

The current study's method was limited in two substantial aspects: the first is the relatively small study group, where a much larger scale incorporating non-student and non-tech-oriented participants should be performed. The second is that the social interaction was only non-verbal, whereas typical human interaction is mainly based on the verbal components. With improved speech recognition and natural language processing technologies, a more natural interaction should be studied.

The current study explored in more fine-grained detail the dynamics of social interaction, as opposed to only pre-post tests. The psychological determinants of moment-by-moment social cognition should be further explored. Moreover, we have expanded on an emerging field of using social robots to study human psychology, such as trustworthiness (DeSteno et al., 2012) and curiosity (Epstein and Gordon, 2018). We suggest that this effective tool has great promise in facilitating more bias-free psychological studies.

## Data Availability Statement

The datasets presented in this study can be found in online repositories. The names of the repository/repositories and accession number(s) can be found below: OSF: https://osf.io/rbvfq.

## Ethics Statement

The studies involving human participants were reviewed and approved by Tel Aviv University Ethics Committee. The patients/participants provided their written informed consent to participate in this study. Written informed consent was obtained from the individual(s) for the publication of any potentially identifiable images or data included in this article.

## Author Contributions

GG developed the study concept. MEp and GG contributed to the study design. Data collection was performed by MEp and MEs and data analysis was performed by MEs and GG. MEs drafted the manuscript. AZ and GG provided critical revisions. All authors approved the final version of the manuscript for submission.

## Conflict of Interest

The authors declare that the research was conducted in the absence of any commercial or financial relationships that could be construed as a potential conflict of interest.
